# Bacillus-related Spore Formers: Attractive Agents for Plant Growth Promotion

**DOI:** 10.1264/jsme2.ME3003rh

**Published:** 2015-09

**Authors:** Koki Toyota

**Affiliations:** 1 Graduate School of Bio-Applications and Systems Engineering, Tokyo University of Agriculture and Technology, 2–24–16 Nakacho, Koganei, Tokyo, 184–8588, Japan

Aerobic spore-forming bacteria, such as *Bacillus* spp., *Paenibacillus* spp., and *Brevibacillus* spp., are one of the major soil bacteria (1, 2, 15). This finding is based on the culture-dependent methods. The culture-independent methods have also revealed that *Bacillus* spp. constitute a majority of the bacteria in a Dutch soil (6) and a soil contaminated with chlorinated compounds in Taiwan (3). In addition to soil communities, *Bacillus* spp. have been isolated from many crops (e.g., 11, 13). *Bacillus* spp. were a dominant bacterial group in the leaves and roots of rice plants; *B. megaterium*, *B. luciferensis*, *Brevibacilllus agri* were isolated from the roots and *B. pumilus*, *B. subtilis*, *Paenibacillus amylolyticus* from the seeds and leaves (12). In a recent study that compared the bacterial community structure of rice plants between leaf blade and leaf sheath, a variety of *Bacillus* spp., including *B. aquimaris*, *B. cereus*, *B. gibsonii*, *B. megaterium*, *B. drentensis*, *B. koreensis*, accounted for 6% to 27% of the total bacterial populations in the leaf blades, while those for 0.6% to 3.3% in the leaf sheaths (10). *Bacillus* spp. and *Paenibacillus* spp. are listed as major components of the bacterial population in the rhizosphere of potato, while *Brevibacillus* spp. are as a minor component (5). The bacterial community of a potato phytosphere was examined using both culture-dependent and independent methods and the results showed that *Bacillus* spp. and *Paenibacillus* spp. composed of 3% to 25% of 16S rRNA gene libraries of the root and tuber, and these ratios were higher than those obtained with the culture-dependent method (21). *Paenibacillus pabuli*, *P. chitinolyticus*, *P. humicus*, and *B. thuringiensis* were isolated from the rhizosphere of agronomic plants as chitinolytic bacteria (20). Metagenomic analysis of the rhizosphere soil of *Lotus japonicas* found that *Firmicutes* constituted 5% of the total bacterial community, although it is not known whether or not they are affiliated with the genus *Bacillus* (24). *Bacillus*-related species have been also isolated from the gut of a soil-feeding termite (16). As described above, *Bacillus*-related species have abilities to colonize a variety of the soil and plant environment. Their most common characteristic is an ability to form endospores and thereby to persist in the environment. Survivability and stress tolerance are always the issues that many microbiologists have tackled for years, and those are now the topic in the context of ecological impacts (8).

While the inoculation of beneficial microbes has attracted attention to enhance crop production (23), the low survivability of the introduced organisms is the drawback in the practical applications. Since spore-forming bacteria are very persistent, the inoculation of *Bacillus* related species to enhance crop growth has a great advantage over non-spore forming bacteria. There are two main mechanisms leading to crop growth promotion: suppression of plant diseases and direct growth promotion. There are growing interests in *Bacillus* related species as biopesticide or biofertilizer.

A recent study revealed that the antimicrobial lipopeptides, iturin A and surfactin, produced by *Bacillus* spp. have a crucial role in the soil-mediated suppression of Fusarium yellows of tatsoi (*Brassica rapa* var. *rosularis*) (25). The compounds were effective in reducing disease severity at low densities without antifungal activity, suggesting the involvement of induced disease resistance in the host plant. It is interesting to note that its disease-suppressing property disappeared by the application of the lipopeptides at higher concentrations. *Bacillus thuringiensis* is a well-known effective bio-insecticide. Some papers have reported *B. thuringiensis* as a biocontrol agent for plant. Strains of *B. thuringiensis* produced acyl homoserine lactone lactonase which opens the lactone ring of *N*-acyl homoserine lactone, a signal molecule in bacterial quorum-sensing system and silences the bacterial virulence (29). There are papers on the productions of a linear aminopolyol antibiotic, zwittermicin A, with high activity against the Oomycetes and their relatives (29) and of bacteriocins with antibacterial activity (4). Recently, Hyakumachi *et al.*(9) revealed that the culture filtrate of *B. thuringiensis* systemically suppressed bacterial wilt of tomato, caused by *Ralstonia solanacearum*, through the systemic activation of the plant defense system, including expression of defenserelated genes such as PR-1, acidic chitinase, and β-1,3-glucanase, in the stem and leaf tissues. *Paenibacillus* strains, isolated from the phyllosphere of tomato, also induced resistance in tomato plants and suppressed Fusarium crown and root rot of tomato, caused by *Fusarium oxysporum* f. sp. *radicis-lycopersici* (18). Among *Bacillus* spp., the number of studies on *B. amyloliquefaciens* is increasing. An iturin and surfactin producing *B. amyloliquefaciens* suppressed the disease severity of damping-off of tomato caused by *Rhizoctonia solani* and bacterial wilt of tomato, although the suppression mechanism was not fully elucidated (26). The following *Bacillus* species have been tested in the field to control bacterial wilt disease: *B. amyloliquefaciens*, *B. methylotrophicus*, *B. subtilis*, *B. vallismortis*, *Brevibacillus brevis* (27). A *Bacillus* strain isolated from the rhizosphere of maize growing in Nigeria showed multiple growth-promoting characteristics consisting of antagonistic activity against *Fusarium verticillioides*, phosphate solubilization efficiency, chitinase activity, and indole-3-acetic acid (IAA) production (1).

Another important feature in *Bacillus* related species is their nitrogen fixing ability. Nitrogen-fixing *Paenibacillus* spp. were isolated from the rhizosphere soils of wheat, corn and susuki (19). Diazotrophic *Bacillus* spp. have been isolated from the rhizosphere of sugarcane (17). A tracer ^15^N_2_ experiment revealed significant biological nitrogen fixation in young sugarcane and showed that most of the fixed nitrogen was distributed in the 80% ethanol insoluble fractions and did not translocate from the root to the shoot, although the exact players in the nitrogen fixation were not fully identified (14). Terakado-Tonooka *et al.*(22) studied the nitrogenase reductase (*nifH*) genes of endophytic diazotrophic bacteria expressed in field-grown sweet potatoes and found that the most dominant clones were derived from *Bradyrhizobium* sp., *Pelomonas* sp., and *Bacillus* sp. In the study, sequences derived from *Bacillus* sp. constituted more than 50% of those amplified from RNA extracted from the stem and tuber of sweet potato (cultivar: beniazuma). A field experiment on young immature oil palm was conducted to quantify the uptake of N derived from N_2_ fixation by diazotrophic *B. sphaericus* UPMB-10, using the ^15^N isotope dilution method, and the results showed that the proportion of N uptake derived from the atmosphere was 63% on the whole plant basis (28). Strains of *B. altitudinis* and *B. pumilus*, originally isolated from the rhizosphere of rice and maize, respectively, formed associations with rice plants and significantly promoted the rice plant growth (7). The performance of the latter strain is proved in many field trials and its formulation and inoculation techniques are under development. The relative abundance of *Bacillus* spp. in rice plants were increased by low nitrogen conditions, suggesting potential contribution of their biological N fixation (10)

To increase crop production due to the growing demands on food supply, we should pay more attention to seek better performing strains and to maximize the abilities of existing strains.
